# Anti-*Helicobacter pylori* antibody status is associated with cancer mortality: A longitudinal analysis from the Japanese DAIKO prospective cohort study

**DOI:** 10.1371/journal.pgph.0001125

**Published:** 2023-02-08

**Authors:** Satoshi S. Nishizuka, Masahiro Nakatochi, Yuka Koizumi, Asahi Hishida, Rieko Okada, Sayo Kawai, Yoichi Sutoh, Keisuke Koeda, Atsushi Shimizu, Mariko Naito, Kenji Wakai

**Affiliations:** 1 Division of Biomedical Research & Development, Iwate Medical University Institute for Biomedical Sciences, Yahaba, Japan; 2 Department of Integrated Health Sciences, Public Health Informatics Unit, Nagoya University Graduate School of Medicine, Nagoya, Japan; 3 Department of Preventive Medicine, Nagoya University Graduate School of Medicine, Nagoya, Japan; 4 Department of Public Health, Aichi Medical University School of Medicine, Nagakute, Aichi, Japan; 5 Division of Biomedical Information Analysis, Iwate Tohoku Medical Megabank Organization, Iwate Medical University, Yahaba, Japan; 6 Department of Medical Safety Science, Iwate Medical University School of Medicine, Yahaba, Japan; 7 Division of Biomedical Information Analysis, Iwate Medical University Institute for Biomedical Sciences, Yahaba, Japan; 8 Department of Oral Epidemiology, Hiroshima University Graduate School of Biomedical and Health Sciences, Hiroshima, Japan; University of Oxford, UNITED KINGDOM

## Abstract

Paradoxically, patients with advanced stomach cancer who are *Helicobacter pylori*-positive (HP^+^) have a higher survival rate than those who are HP^-^. This finding suggests that HP infection has beneficial effects for cancer treatment. The present study examines whether HP^+^ individuals have a lower likelihood of death from cancer than those who are HP^-^. Prospective cohort data (*n* = 4,982 subjects enrolled in the DAIKO study between 2008–2010) were used to assess whether anti-HP antibody status was associated with cancer incidence. The median age in the primary registry was 53 years-old (range 35–69 years-old). Over the 8-year observation period there were 234 (4.7%) cancer cases in the cohort and 88 (1.8%) all-cause deaths. Urine anti-HP antibody data was available for all but one participant (n = 4,981; 99.98%). The number of HP^+^ and HP^-^ individuals was 1,825 (37%) and 3,156 (63%), respectively. Anti-HP antibody distribution per birth year revealed that earlier birth year was associated with higher HP^+^ rates. With a birth year-matched cohort (*n* = 3,376), all-cancer incidence was significantly higher in HP^+^ individuals than those who were HP^-^ (p = 0.00328), whereas there was no significant difference in the cancer death rate between HP^+^ and HP^-^ individuals (p = 0.888). Cox regression analysis for prognostic factors revealed that the hazards ratio of HP^+^ was 1.59-fold (95%CI 1.17–2.26) higher than HP^-^ in all-cancer incidence. Potential systemic effects of HP^+^ status may contribute to reduced likelihood of death for patients after an initial diagnosis of cancer.

## Introduction

*Helicobacter pylori* (HP) infection is considered to be an etiologic carcinogen of the stomach [1]. Paradoxically, however, investigators have recognized that the survival rate of patients with advanced gastric cancer who are HP^+^ is higher than that for HP^-^ patients from studies carried out in a variety of countries that have varying HP genotype and diet as well as different therapeutic strategies for advanced gastric cancer [2–8]. Regarding HP infection in the context of post-operative adjuvant chemotherapy for patients with Stage II/III gastric cancer [9,10], our previous study demonstrated that those patients who were HP^+^ had a clearly higher survival rate over the 10-year observation period [11]. In particular, patients who were HP^+^ and received adjuvant chemotherapy with S-1 showed >20% better overall survival than did those who were HP^-^, which was further confirmed by propensity score matching analysis. Stratified Cox proportional hazards regression analysis revealed that all 10 subgroups had better survival in the respective HP^+^ groups [11]. More recently, our separate analysis revealed that the better survival rate in HP^+^ patients was further pronounced in patients with PD-L1^-^ as well as S-1 adjuvant chemotherapy [12]. These results collectively suggest that HP infection sustains potential anti-tumor host immunity, which may contribute to prolonged survival.

Alternatively, HP infection could trigger non-specific enhancement of innate immune system activity to create a *de facto* heterologous immunological memory that has been termed trained immunity [13]. Such triggered immunity that has some anticancer effects has been shown in patients with high-risk non-muscular invasive bladder cancer for which intravesical instillation of Bacillus Calmette-Guérin (BCG) has been the gold-standard treatment [14]. For gastric cancer, immunotherapy using OK-432, a freeze-dried product prepared by incubating the low-virulence Su strain of group A *Streptococcus pyogenes* with penicillin [15], has been actively investigated [16]. Although not the gold-standard for gastric cancer, therapies like OK-432 that promote immune responses appear to suppress relapse in both humans and mouse models of gastric cancer [17–20]. If immune activation indeed plays a role in suppressing either the development or progression of cancer, then sustained HP infection should also show some beneficial effect in terms of cancer incidence and all-cause or cancer death in a longitudinal cohort.

The subject patients of advanced gastric cancer, including ours, had undergone gastrectomy, meaning that HP could no longer colonize the stomach. Thus, the observed effect of HP on prolonged survival could be systemic and perhaps lifelong, and thus may have an antitumor potential. Using data from the DAIKO prospective cohort in Nagoya, Japan, the present study aimed to understand whether the putative effect associated with HP infection has an antitumor effect with respect to reduction of incidence of all-cancer as well as breast, colorectal, lung, prostate, and stomach cancers. The effects of HP on all-cancer deaths were also assessed. Although the observation period of the DAIKO cohort to date is eight years, the beneficial effect of HP infection can be determined if the all-cancer death rate in HP^+^ individuals is lower than the all-cancer incidence in those who are HP^+^. This putative effect suggests that HP^+^ cancer patients have a lower likelihood of death compared to HP^-^ cancer patients.

## Materials and methods

### Ethics statement

Ethical approval for the parent Japan Multi-Institutional Collaborative Cohort Study (J-MICC) study was granted by Nagoya University Graduate School of Medicine Institutional Review Board [21]. Based on the IRB approval by the Nagoya University Graduate School of Medicine, a separate ethical approval for the current analysis using the DAIKO study was granted by both Nagoya University Graduate School of Medicine IRB (2008-0618-2) and Iwate Medical University School of Medicine IRB (MH2018-019). The study was conducted according to Helsinki Declaration principles.

### Source cohort

The DAIKO study includes 5,165 participants aged 35–69 years-old who were residents of Nagoya and were enrolled between June 2008 and May 2010 at the Daiko Medical Center of Nagoya University, Nagoya, Japan. The J-MICC Study is the parental cohort of the DAIKO study, which includes 13 institutes across Japan with enrollment of >100,000. Follow-up surveys have been conducted since 2005 [21]; and the end of follow-up of the current study was Dec 31, 2016. The main objective of the J-MICC study is to investigate gene-environmental interactions with lifestyle-related diseases, mainly cancer. Recruitment for the DAIKO study was conducted as a part of the J-MICC study. The subject sources for J-MICC were as follows: 1) volunteers residing in the areas defined by local governmental administration; 2) health checkup examinees run by local governments; 3) visitors of health checkup facilities; and 4) visitors of a cancer hospital [21], whereas only the subject source of 1) was applied for the DAIKO study. In addition, the following criteria were applied for the DAIKO subject: 1) age between 35–69 years for males or females; and 2) residents of Nagoya city. The participants were invited by distribution of leaflets to the residents in Nagoya city. The DAIKO study is not a patient cohort, but may have included cancer patient at baseline during registration. The cohorts have also provided opportunities to perform cross-sectional studies on lifestyle factors, biomarkers, and genotypes [22–26]. In the present study, we analyzed DAIKO study data in terms of cancer incidence and survival to verify whether these events were associated with anti-HP antibody status that indicates the immune status of an individual with respect to past/present HP infection. Although the current analysis of this paper focuses on cancer incidence and deaths, due to the nature of the non-patient individual cohort, we do not have access to detailed patient information, such as staging, treatment, and pathological and genomic status.

### Study population

Participants in the present study were a subset of the DAIKO study, from whom written informed consent was obtained upon enrollment in studies outside of the J-MICC-assigned program. Baseline data were obtained from 5,165 participants, and 183 participants were excluded for the following reasons: lack of consent to be a part of research studies except for the parent J-MICC study (n = 170); ineligibility (n = 12); and revocation of informed consent at baseline (n = 1). Overall, 4,982 (96.5% of the J-MICC study participants) individuals were enrolled in the present study.

### Baseline variables

Baseline covariates of the present study included age, birth year, gender, body mass index, blood pressure, laboratory blood data such as total cholesterol, high-density lipoprotein cholesterol, aspartate aminotransferase, history of cancer, history of medication for HP, and urine anti-HP antibody status determined using an immunochromatography kit (RAPIRAN, Otsuka Pharmaceutical, Tokushima, Japan) [27–29]. For the baseline laboratory blood data analysis, peripheral blood was drawn in the morning using three 7-ml vacuum tubes after overnight fasting. Details of the procedures were described in our previous study [30]. The RAPIRAN is a *de facto* standard kit for anti-HP antibody detection in urine [31]. It has been reported that the sensitivity, specificity, and consistency of the RAPIRAN kit against an independent serum enzyme immunoassay E-plate (Eiken, Tokyo, Japan) was 86.2%, 93.7%, and 90.1%, respectively [31,32]. Moreover, an apparent birth-year effect to HP infection rate can be assessed in order to evaluate if the RAPIRAN kit has introduced a bias for anti-HP antibody detection in the current cohort.

### Outcome measures

The primary end point was all-cancer incidence, and all-cause death. The all-cancer incidence was further categorized into cancer types when appropriate. Analysis of individual cancer types included those of asynchronous secondary cancer. The classification of cancer types was defined using the International Statistical Classification of Diseases for Oncology, Third Edition (ICD-O-III). The vital and residential status of participants was determined by using the population registry. For logistical reasons, we ended the follow-up of subjects who had moved out of the study area (Nagoya city). Causes of death were confirmed based on the vital statistics data provided by the Ministry of Health, Labor and Welfare, Japan. Cancer incidence data was obtained from the Aichi Cancer Registry.

### Statistical analysis

In principle, all variables were compared based on the anti-HP antibody status defined in a binary manner (i.e., presence of antibody, HP-positive, HP^+^; absence of antibody, HP-negative, HP^-^). Categorical and continuous variables were analyzed using Fisher’s exact test and Wilcoxon’s rank sum test, respectively. Subject matching was performed to correct for birth year.

Crude rates of cancer incidence and death are shown as the number of cancer incident cases and deaths per 1,000 person-years, respectively. The crude rates were compared between HP^+^ and HP^-^ using the Wald test.

To minimize the effects of a lack of background uniformity when comparing cancer incidence/death and anti-HP antibody status, the following procedure was performed when necessary: (a) Exclusion of participants who had a case history of any type of cancer; and (b) frequency matching according to birth year by gender. The proportion of event-free survival was estimated using the Kaplan-Meier method. A log-rank test was used to examine the null hypothesis whereby there was no difference in the proportion of event-free survival between the populations in terms of the probability of an event at any time point. A Cox proportional hazards model was used to estimate the hazards ratio (HR). The follow-up period was computed from the baseline survey to cancer incidence (in the analysis for cancer incidence), death of any cause, moving out of the study area, and the end of follow-up (Dec 31, 2016). The model included age, sex (1: male, 2: female), smoking status (1: current or ever, 0: never), and drinking habits (1: current or ever, 0: never). P-value <0.05 was considered statistically significant. All statistical analyses were performed using R.

## Results

### Participant characteristics

The purpose of the parental cohort, the J-MICC study, was to confirm and detect gene-environment interactions of lifestyle-related diseases, mainly of cancer, through the cohort analysis. It included a cross-sectional analysis of lifetime factors, biomarkers, and genotypes as well as confirmation/screening of new biomarkers that can be used for early diagnosis of cancer [21]. In the present study, however, we exclusively focused on investigating whether HP infection status was associated with an all-cancer death rate. The population of the DAIKO cohort study that was eligible for the present study (n = 4,982) included 1,416 men and 3,566 women, with a median age of 53. Results of urine anti-HP antibody tests were available for 4,981 out of 4,982 (99.98%) participants. There were 1,825 (37%) HP^+^ individuals and 3,156 (63%) HP^-^ individuals. The summary of participant characteristics is shown in **Table 1**. Individuals who were HP^+^ tended to be male and older relative to HP^-^ individuals. HP^+^ status was also associated with higher BMI and history of smoking. Although the absolute difference was small, all clinical and laboratory continuous variables tested exhibited higher risk factors for major chronic diseases for HP^+^ compared to HP^-^ individuals (S1 Table) [33,34].

**Table 1:** Characteristics of study participants.

		HP^+^ (*n* = 1,825)		HP^-^ (*n* = 3,156)		
Variable	Value	*N*	%	*n*	%	*P* value
Sex	Male	562	30.8	854	27.1	0.00506
	Female	1,263	69.2	2,302	72.9	
Age	Median(1st-3rd quartile)	58.6 (49.3, 64.7)		50.3 (42.4, 60.5)		3.47 x 10^−60^
BMI^a^	Median(1st-3rd quartile)	21.5 (19.7, 23.8)		21.1 (19.4, 23.3)		0.0000874
Smoking^b^	Yes	609	33.4	962	30.5	0.034
	No	1,215	66.6	2,194	69.5	
Drinking	Yes	1,009	55.3	1,795	56.9	0.273
	No	816	44.7	1,360	43.1	

HP, *Helicobacter pylori*; BMI, Body mass index; Yes, current or ever; No, never

^a^HP^-^ (*n* = 3,155)

^b^HP^+^ (*n* = 1,824).

### HP eradication

HP is classified as a Group 1 carcinogen by the World Health Organization. Symptoms frequently involved in HP infection include nausea, epigastralgia, and heartburn. These symptoms may ultimately be considered to be triggers for peptic ulcers and gastric cancer. To reduce these symptoms and risk, HP eradication therapy has been conducted in Japan [35]. The chronological change in anti-HP antibodies has been considered to be a potential predictor of successful HP eradication therapy, although the immunological significance of antibody titer remains to be elucidated [36]. A recent study from a large-scale cohort study in Japan suggested that the majority of patients who received eradication therapy had reduced anti-HR antibody levels within one year [36]. In the present study, we compared the measured urine anti-HP antibody levels and self-reported HP eradication history. Nearly 90% of participants had never been assessed for HP prior to enrolling in the DAIKO study. Therefore, the majority of participants reflect the current HP infection status. Among the 472 (9.5%) participants who had undergone an HP examination (method unknown), 314 (66.5%) were positive, and 133 (28.2%) were negative. The status of 25 individuals (5.3%) was unknown or no credible information available. Among the previously-diagnosed HP^+^ participants (n = 314), 152 (48.4%) were anti-HP antibody positive (HP^+^) while 162 (51.6%) were HP^-^ at the DAIKO baseline. Among the previously-diagnosed HP^-^ participants (n = 133), 26 (19.5%) were HP^+^ while 107 (80.5%) were HP^-^ at DAIKO baseline. Thus, the consistency rate was 57.7% among any previous HP exam and the present urine anti-HP antibody test. It should be noted that HP^-^ participants at the DAIKO baseline could include those for whom HP was successfully eradicated. In fact, among the previously-HP^+^ participants, 162 (51.6%) had successful eradication. The self-claimed “successful eradication” resulted in 53 (33.1%) anti-HP antibody positive subjects, whereas 109 (66.9%) were negative at baseline. No definitive time point for eradication was available for the present cohort. Of note, if the eradication therapy was conducted within one year, one to five years, and more than 6 years before the cohort enrollment, it was reported that anti-HP antibody titer was decreased by 76.8%, 88.2%, and 91.5%, respectively [36]. Furthermore, information about the antibiotics used for the eradication was not available.

### Birth year effect

**Fig 1:**
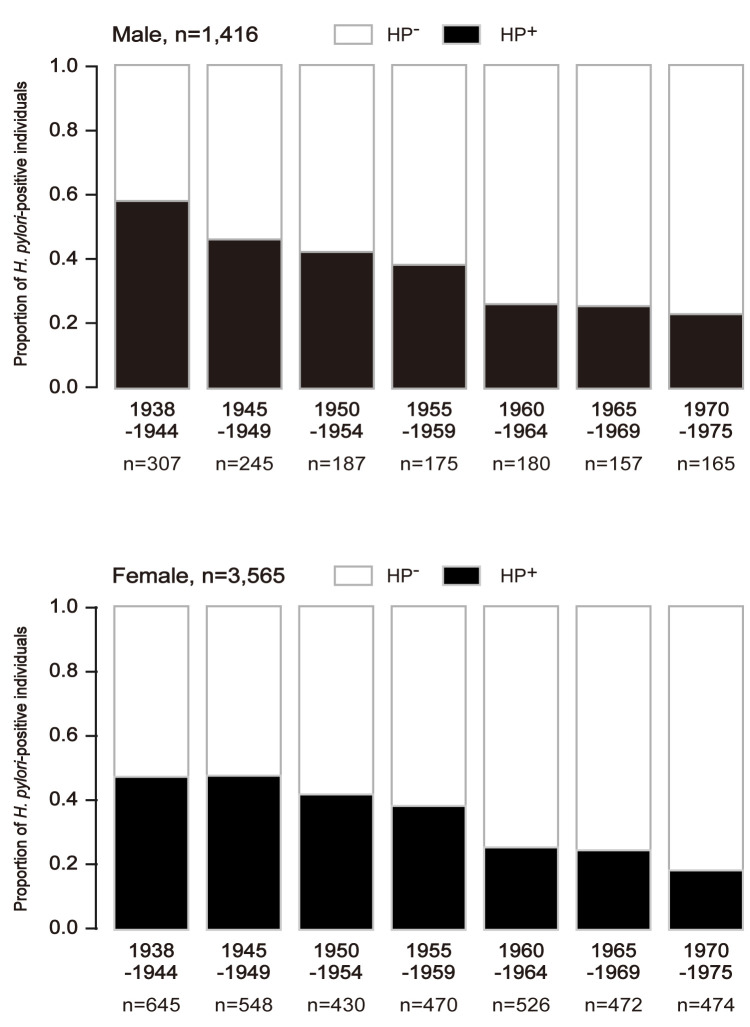
Anti-HP antibody status by birth year. The HP^+^ rate is shown with five-year bins for male participants (upper panel, n = 1,416: 1938–1944, n = 307; 1945–1949, n = 245; 1950–1954, n = 187; 1955–1959, n = 175; 1960–1964, n = 180; 1965–1969, n = 157; 1970–1975, n = 165) and female participants (bottom panel, n = 3,565; 1938–1944, n = 645; 1945–1949, n = 548; 1950–1954, n = 430; 1955–1959, n = 470; 1960–1964, n = 526; 1965–1969, n = 472; 1970–1975, n = 474) according to birth year. Black areas in each bar represent the HP^+^ fraction.

In addition to biological/epidemiological information, the birth-year effect on HP infection rate may confirm that the reliability of the HP infection detection kit (i.e., RAPIRAN) was reasonable. It is known that the HP infection rate in the general population has been decreasing since the 1940s [37]. We thought the birth-year HP infection rate bias may be substantial, and therefore it should be used for statistical matching. The number of HP^+^ individuals categorized by birth year was 76 (1.5% of all HP^+^ cases) for 1938–1939, 876 (17.6%) for 1940–1944, 793 (15.9%) for 1945–1949, 617 (12.4%) for 1950–1954, 645 (12.9%) for 1955–1959, 706 (14.2%) for 1960–1964, 629 (12.6%) for 1965–1969, 630 (12.6%) for 1970–1974, and 9 (0.18%) for 1975. There was a clear trend that lower birth year was associated with higher rate of HP^+^ (**Fig 1**). The mean HP^+^ fraction by birth year 1938–1944 was 0.58, whereas that for 1970–1975 was 0.21. The fraction of females with HP^+^ and birth year 1938–1944 was 0.45, whereas that for 1970–1975 was 0.15. The HP^+^ fraction for men was higher than that for women across the birth years. The present data seem consistent with previous data from the Japanese population [37]. Overall, we conclude that information obtained from the RAPIRAN kit was reasonable with minimal bias.

### Matching

Matching is a technique used to avoid confounding in a study design. The HP^+^ participants were frequency-matched to HP^-^ respondents for five-year bins of birth year and gender to minimize confounding factors. Those subjects who had a previous history of cancer were excluded. With birth year matching, the categorical patient characteristics showed no significant difference between HP^+^ and HP^-^ individuals (**Table 2**). Moreover, after matching, almost all continuous variables showed no difference between HP^+^ and HP^-^ individuals except for the HDL cholesterol level for which the estimated mean difference was 3 mg/dl (S2 Table).

**Table 2:** Characteristics of participants after matching.

		HP^+^ (*n* = 1,688)		HP^-^ (*n* = 1,688)		
Variable	Value	*n*	%	*n*	%	*P* value
Sex	Male	489	29.0	489	29.0	1.00
	Female	1,199	71.0	1,199	71.0	
Age (yrs)	Median(1st-3rd quartile)	57.9(48.6, 64.1)		57.8(48.8, 63.9)		0.866
BMI^a^	Median(1st-3rd quartile)	21.5(19.7, 23.8)		21.4(19.7, 23.5)		0.37
Smoking^b^	Yes	545	32.3	522	30.9	0.395
	No	1,142	67.7	1,166	69.1	
Drinking	Yes	920	54.5	968	57.3	0.103
	No	768	45.5	720	42.7	

HP, *Helicobacter pylori*; BMI, Body mass index; Yes, current or ever; No, never

^a^HP^-^ (*n* = 1,687)

^b^HP^+^(*n* = 1,687).

### Cancer incidence

Using the data generated by matching, we first analyzed cancer incidence tabulated with HP infection status. It is clear that gastric cancer has the highest impact from HP infection, so we analyzed individuals with gastric and non-gastric cancer in the present cohort. The non-gastric cancer types were chosen based on the number of incidents that are relatively large among available data sets. Overall cancer incidence was 105 and 67 in HP^+^ (n = 1688) and HP^-^ (n = 1688) individuals, respectively (p = 0.003, Wald test). The individual cancer types that had significantly higher incidence in the HP^+^ population were both gastric (20 and 5 in HP^+^ and HP^-^ individuals, respectively) and non-gastric (87 and 62 in HP^+^ and HP^-^ individuals, respectively). Other cancer types including uterine, lung, prostate, colon, and breast showed no significant difference in cancer incidence in terms of HP infection status, although the number of cases was too small to reach a definitive conclusion about the effect of HP status. The all-cause death incidence in terms of HP status also showed no significant difference. The cancer incidence after matching is shown in S3 Table. These results suggest that HP infection may increase the risk for a wide range of cancer, although the risk for gastric cancer is more pronounced than other cancer types.

### Survival analysis

**Fig 2:**
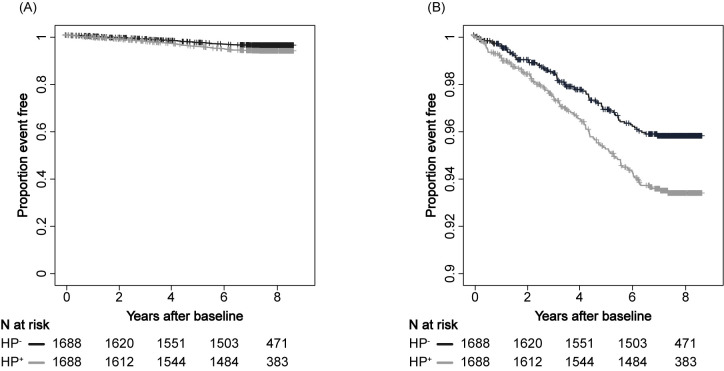
All cancer incidence. Kaplan-Meier estimation of all cancer incidence detected in this study (i.e., gastric, uterine, lung, prostate, colon, and breast cancer) stratified by anti-HP antibody status. The black line indicates HP^+^ and the gray line indicates HP^-^. Panel B is a partial enlarged view of panel A. A, vertical axis range 0.00–1.00; B, vertical axis range 0.90–1.00.

We next analyzed the cancer incidence and death rate using the Kaplan-Meier method. The crude cumulative all-cancer incidence rate was higher for HP^+^ individuals than that for HP^-^ (n = 3,376, P = 0.00328; **Fig 2**).

**Fig 3:**
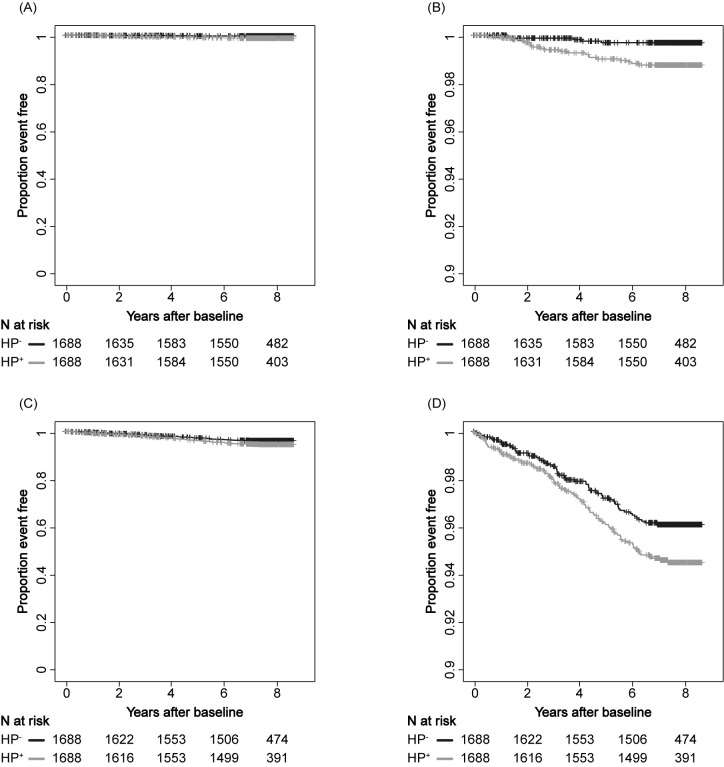
Gastric and non-gastric cancer incidence. Kaplan-Meier estimation of gastric (A, B) and non-gastric (C, D) cancer incidence stratified by anti-HP antibody status. Non-gastric cancer includes all those who started with ICD-O-III (i.e., not limited to uterine, lung, prostate, colon, and breast cancers). The black line indicates HP^+^ and the gray line indicates HP^-^. Panels B and D is a partially enlarged view of panels A and C, respectively. A and C, vertical axis range 0.00–1.00; B and D, vertical axis range 0.90–1.00.

As expected, the incidence of gastric cancer was significantly higher in HP^+^ individuals than those who were HP^-^ (p = 0.0027; **Fig 3A and 3B**). Interestingly, however, the incidence of non-gastric cancer was also significantly higher in HP^+^ individuals than those who were HP^-^ (p = 0.039; **Fig 3C and 3D**). Consistent with the above tabulated analysis, this result suggests that HP infection may increase the risk for a wide range of cancers, although the risk for gastric cancer is more pronounced than other cancer types. The cumulative cancer incidence was also assessed for lung, colorectal, rectum, prostate, and breast cancer, although the number was limited (**S1 Fig**).

**Fig 4:**
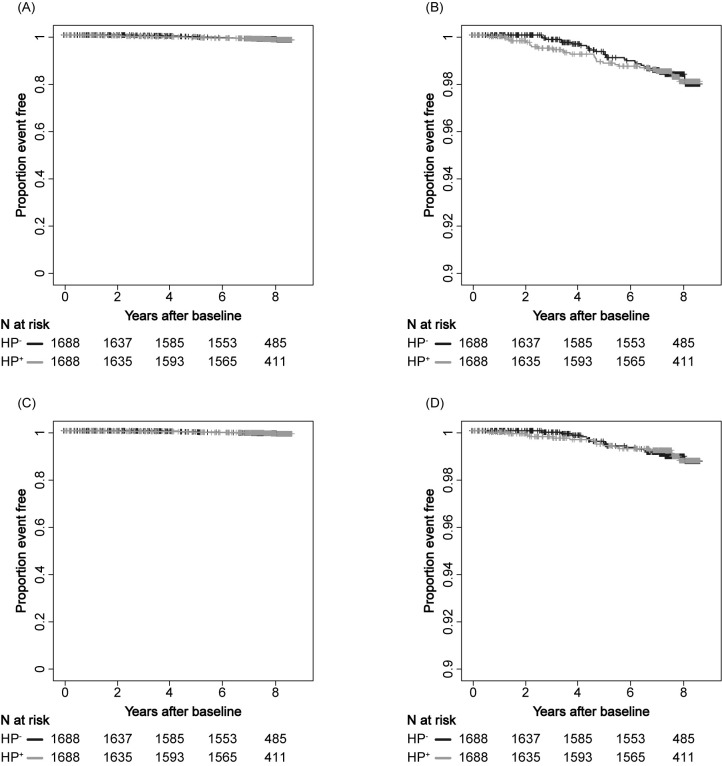
All-cause and cancer-specific deaths. Kaplan-Meier estimation of all-cause deaths (A, B) and deaths due to cancer (C, D) stratified by anti-HP antibody status. Cancer type includes gastric, uterine, lung, prostate, colon, and breast. The black line indicates HP^+^ and the gray line indicates HP^-^. Panels B and D are partially enlarged views of panels A and C, respectively. A and C, vertical axis range 0.00–1.00; B and D, vertical axis range 0.90–1.00.

The cumulative all-cause death rate was not significantly different between HP^+^ and HP^-^ individuals (p = 0.972; **Fig 4A and 4B**). It is important to note that the cancer incidence was higher in the HP^+^ population than the HP^-^ population for both gastric and non-gastric cancer. Therefore, the finding that there was no significant difference between the HP^+^ and HP^-^ populations in overall survival suggests that the HP^+^ population has a lower risk for death than that of HP^-^ population. In addition, death related to cancer almost always occurs in advanced stages, whereas cancer incidence is generally measured at early stages. Therefore, the deceased population should have received some treatment procedures. One possible hypothesis is that potential systemic effects of HP infection may contribute to a reduced likelihood of death in the context of advanced cancer treatment. Although a limited number of deaths were recorded (n = 33 out of 3,376 matching cases), cancer-specific deaths did not differ between HP^+^ and HP^-^ individuals (n = 3,376, p = 0.888; **Fig 4C and 4D**).

To identify independent predictive variables for the risk of incidence for all-cancer, gastric, and non-gastric cancer, a Cox proportional-hazards model was applied with variables including age, sex, drinking, smoking, and HP infection. The hazards ratio of all-cancer incidence by HP was 1.59 (p = 0.00297), which was lower than the hazard ratio for smoking (1.97; p = 0.000422) but higher than that for alcohol consumption (1.24; p = 0.216, S4 Table). The hazards ratio for the gastric cancer incidence was 3.93 (p = 0.00631, S5 Table), whereas that for non-gastric cancer incidence in HP^+^ over HP^-^ was 1.42 (p = 0.0356, S6 Table). However, the number of gastric cancer cases was 20 and 5 in HP^+^ (n = 1,688) and HP^-^ (n = 1,688) individuals, respectively, which was too small to allow additional modeling with respect to other cancer types in the current study observation period.

## Discussion

In our study cohort from the DAIKO study, we demonstrate that the cancer incidence of the HP^+^ population was significantly higher than the HP^-^ population. However, the all-cancer death rate for the HP^+^ population was similar to that for the HP^-^ population. Two possible hypotheses can be drawn from these observations. First, the HP^+^ population appears to be more susceptible to cancer. Second, HP^+^ populations may have higher response or sensitivity to treatment for advanced cancer. Important premises can explain these hypotheses including establishment of HP^+^ infection early in life [38–41] and therapy for advanced cancer in Japan is almost always attempted before death as part of coverage by the Japanese universal health insurance system, *kaihoken* [42]. Therefore, the present results suggest that HP infection may have triggered acquisition of innate immunity that is beneficial for advanced cancer therapy [13].

However, the notion that innate immunity is ultimately relevant to death and plays a role in advanced cancer treatments has been poorly presented to date. In the present study, non-gastric cancer types, including (but not limited to) uterine, lung, prostate, colon, and breast were studied to evaluate whether HP infection was associated with cancer incidence as well as death rate. In fact, there has not been definitive evidence directly linking HP infection with cancer death [43–47]. Due to limitations on data access in this study, we only showed that the HP^+^ population had a higher cancer incidence rate than the HP^-^ population, whereas there was no difference in the death rate. These results suggest that HP^+^ population has a lower risk for death after the initial diagnosis of cancer. Importantly, almost all cancer patients are treated after diagnosis, and therefore the HP^+^ population may have better treatment outcomes. Although it remains to be elucidated if HP infection may influence treatment efficacy, it may be of a practical hallmark to stratify treatment indications.

After recognition of a pathogen, one of the ultimate purposes of disease control is to reduce deaths caused by the pathogen. For gastric cancer, eradication of HP has been considered to reduce a variety of gastric diseases including gastritis, peptic ulcer, and potentially gastric cancer [48–50]. However, the role of HP in gastric carcinogenesis may be limited to the development of “early” gastric cancer, in which normal mucosal cells transform to a malignant phenotype [51,52]. More advanced-stage gastric cancer cases have substantial invasion beyond the submucosae where cancer cell division is no longer regulated by the malignant signal transduction induced by HP, which has been well presented in the “hit-and-run” model [53].

In Japan, the 5-year disease-free survival of pT1N0M0 gastric cancer is 99%, and that for pT1N1M0/pT2N0M0 is 94% and 92%, respectively [54]. Thus, early gastric cancer may not be prioritized in terms of efforts to reduce gastric cancer-deaths. It is clear that nearly all gastric cancer-deaths are caused by advanced stage or recurrent gastric cancer. Therefore, the beneficial effect of HP in these gastric cancer patients should be considered differently from the simple malignant transformation of gastric mucosal cells. Here we suggest that stratification of treatment for these advanced, including non-gastric, cancer patients by HP infection status in which promoting therapy or maintaining alternative regimens for the HP^-^ population might be a prompt practical option.

The prevalence of HP in some regions of the world remains high [55]. The eradication of HP appears to be associated with a reduced incidence of gastric cancer [56]. It is, however, important to consider the fact that gastric cancer death rate has been continuously decreasing in many ethnic groups and countries including Japan since 1970s, without a concerted nationwide eradication program [37,57–60]. In the present study, we demonstrated that anti-HP antibody status was associated with birth year in that those having later birth year had a lower HP^+^ rate. These findings suggest that the role of HP in cancer treatment is one of the treatment stratification indices for advanced gastric cancer patients in most countries, whereas the number of gastric cancer deaths will likely continue to decrease naturally without efforts for HP eradication.

The present study has some limitations. The observational period is eight years, and the mean age at baseline was 58. A longer observation period would increase the cancer incidence and number of deaths. In fact, the number of all-cancer deaths may be too small to be conclusively analyzed as an independent parameter. We would have more evaluable cases in terms of tumor-type specific HP effect other than gastric cancer. The HP eradication history was reported by individual participants as personal medical records were not fully accessible for the present cohort. The HP infection was evaluated by only testing for anti-HP antibody in the urine. The anti-HP antibody status may be considered to reflect past or present HP infection, but no information about whether urine antibody levels had naturally attenuated was available. Therefore, this parameter might be less robust than extracting positive results from multiple HP examinations. However, past reports for HP infection rate that took birth-year into account were consistent with our birth-year effect observations (i.e., earlier birth year was associated with higher HP^+^ rates). Although our current HP infection status definition could have been affected by our detection kit (i.e., RAPIRAN kit), this consistency supports that the anti-HP antibody detection method using the RAPIRAN kit may have minimized the possible bias to the actual HP infection status. The HP strain was not assessed, although most HP strains in Japan are CagA^+^ [61]. Depending on the practicality and objectives, detailed characterization of HP may be warranted in future studies. Moreover, the cohort subject is based on non-patient individuals, not a patient cohort. Hence, information such as treatment details, cancer stage, and molecular characteristics were not available.

Although details on the participants (i.e., performance status) were not accessible, the present finding has been well supported by our recent study demonstrating that HP^+^ post-gastrectomy patients whose Programmed Death-Ligand1 (PD-L1) status was negative had significantly better survival than those who were HP^-^ after adjuvant chemotherapy using S-1 [12]. That study was limited to advanced gastric cancer patients, but a systemic effect may have been involved in the treatment response. Therefore, our current results showing that the HP^+^ population had better overall survival may be reasonably applied to multiple cancer types.

HP infection is associated with susceptibility to diverse types of cancer. However, HP infection has a favorable survival effect for patients with advanced cancer. With previous reports showing a better prognosis of advanced gastric cancer patients who are HP^+^ than those for HP^-^ patients from various regions of the world [2–8,11] where the therapeutic regimens are diverse, HP infection may be a predominant factor for survival of not only patients with gastric cancer, but also for those with other types of cancer. In conclusion, our present results may suggest that HP infection may reduce the risk for death after an initial diagnosis of cancer.

## Supporting information

S1 Fig Individual cancer incidence Kaplan-Meier estimation of individual cancer incidence stratified by anti-HP antibody status. A, Lung cancer; B, Colorectal cancer; C, Rectal cancer, D, Prostate cancer; and E, Breast cancer. Left, axis range 0.00–1.00; right, axis range 0.90–1.00. The subject patients were male only (prostate) and female only (breast).
(DOCX)

S1 Table Continuous variables for the participants. (DOCX)

S2 Table Continuous variables for participants after matching. (DOCX)

S3 Table Cancer incidence rate and all-cause deaths. (DOCX)

S4 Table Multivariate Cox regression models for all-cancer incidence (*n* = 3,375). (DOCX)

S5 Table Multivariate Cox regression models for gastric cancer incidence (*n* = 3,375). (DOCX)

S6 Table Multivariate Cox regression models for non-gastric cancer incidence (*n* = 3,375). (DOCX)

## Abbreviations

HP: *Helicobacter pylori*
CI: confidence interval
J-MICC: Japan Multi-Institutional Collaborative Cohort Study
