# Supplementary material for: Anti-Helicobacter pylori antibody status is associated with cancer mortality: A longitudinal analysis from the Japanese DAIKO prospective cohort study
Source: PLOS Glob Public Health. 2023 Feb 8;3(2):e0001125. doi: 10.1371/journal.pgph.0001125 (PMC10022139; doi:10.1371/journal.pgph.0001125)
Supplement: S1 Fig — A, Lung cancer; B, Colorectal cancer; C, Rectal cancer, D, Prostate cancer; and E, Breast cancer. Left, axis range 0.00–1.00; right, axis range 0.90–1.00. The subject patients were male only (prostate) and female only (breast). (DOCX) [file pgph.0001125.s001.docx]

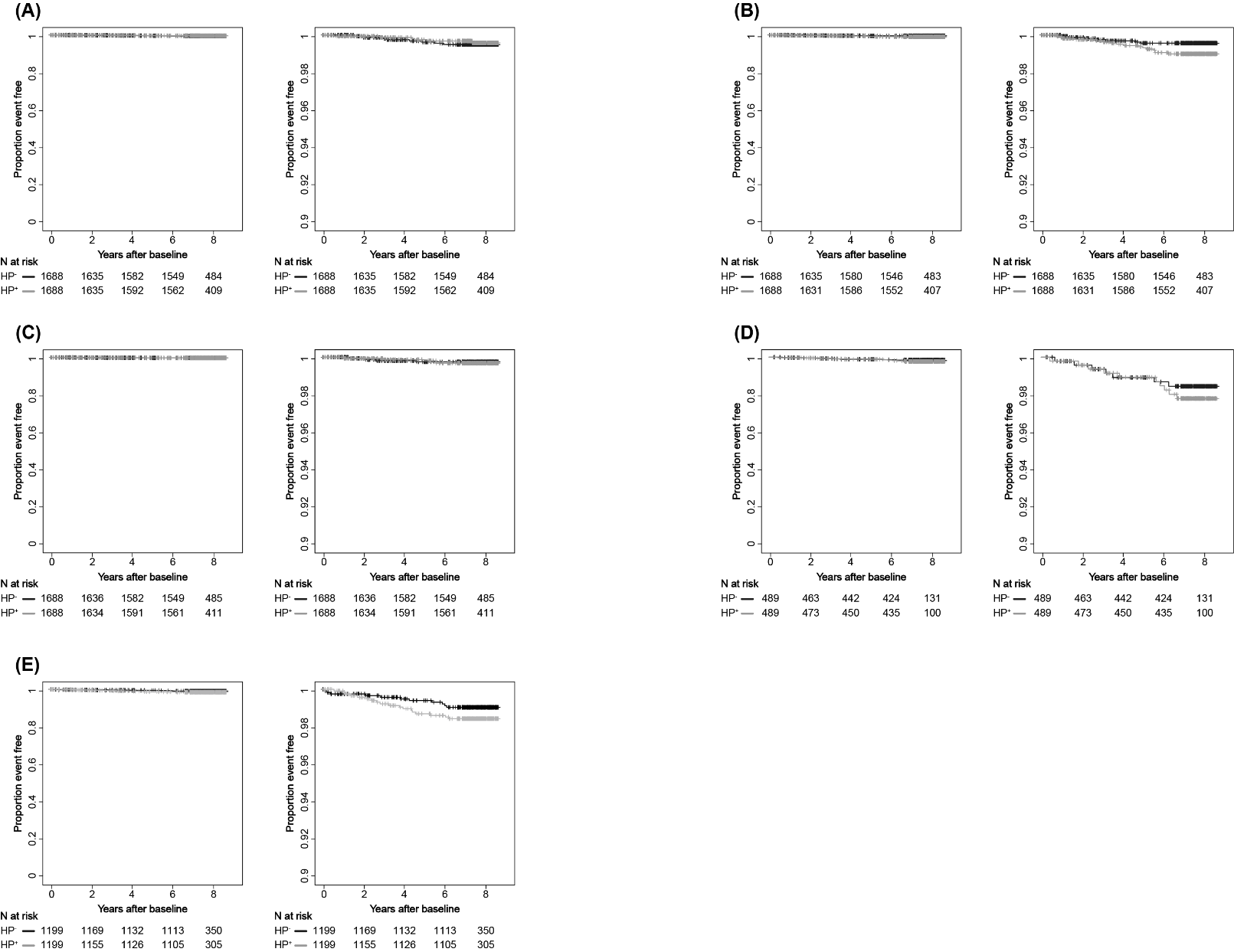


**S1 Fig Individual cancer incidence** Kaplan-Meier estimation of individual cancer incidence stratified by anti-HP antibody status. A, Lung cancer; B, Colorectal cancer; C, Rectal cancer, D, Prostate cancer; and E, Breast cancer. Left, axis range 0.00-1.00; right, axis range 0.90-1.00. The subject patients were male only (prostate) and female only (breast).
