# Supplementary material for: Anti-Helicobacter pylori antibody status is associated with cancer mortality: A longitudinal analysis from the Japanese DAIKO prospective cohort study
Source: PLOS Glob Public Health. 2023 Feb 8;3(2):e0001125. doi: 10.1371/journal.pgph.0001125 (PMC10022139; doi:10.1371/journal.pgph.0001125)
Supplement: S1 Table — (DOCX) [file pgph.0001125.s002.docx]

**S1 Table** **Continuous variables for the participants**

| Variable (unit) | HP^+^(*n*=1,825)  Median(1st-3rd quartile) | HP^-^(*n*=3,156)  Median(1st-3rd quartile) | *P* value |
| --- | --- | --- | --- |
| Age (yr) | 58.6 (49.3, 64.7) | 50.3 (42.4, 60.5) | 3.47 x 10^-60^ |
| Waist (cm)^a^ | 80.8 (74.8, 87) | 78.6 (72.8, 85) | 6.04 x 10^-12^ |
| SBP (mmHg) | 117.5 (105.5, 133) | 113.5 (103, 127.5) | 3.24 x 10^-12^ |
| DBP (mmHg) | 71.5 (64, 81.5) | 70.5 (63, 79.5) | 0.000289 |
| TC (mg/dL)^b^ | 210 (188, 235) | 205 (182, 229) | 5.91 x 10^-8^ |
| TG (mg/dL)^b^ | 83 (57.8, 119) | 74 (54, 109) | 5.87 x 10^-8^ |
| HDL (mg/dL)^b^ | 61 (52, 73) | 65 (55, 76) | 2.60 x 10^-10^ |
| AST (GOT) (IU/L)^b^ | 20 (17, 24) | 19 (17, 23) | 2.68 x 10^-6^ |
| ALT (GPT) (IU/L)^b^ | 16 (12, 21) | 15 (11, 20) | 0.00893 |
| γ−GTP (IU/L)^b^ | 20 (14, 32) | 18 (13, 29) | 6.15 x 10^-6^ |
| Cr (mg/dL)^b^ | 0.6 (0.6, 0.7) | 0.6 (0.6, 0.7) | 0.00703 |
| UA (mg/dL)^b^ | 4.7 (4, 5.7) | 4.5 (3.8, 5.5) | 4.26 x 10^-7^ |

HP, *Helicobacter* *pylori*; BMI, body mass index; SBP, systolic blood pressure; DBP, diastolic blood pressure; TC, total cholesterol; TG, triglycerides; HDL, high-density lipoprotein cholesterol; AST (GOT), aspartate aminotransferase (glutamic oxaloacetic transaminase); ALT (GPT), alanine aminotransferase (glutamic pyruvic transaminase). γ−GTP, gamma-glutamyl transpeptidase; Cr, creatinine; UA, uric acid; ^a^HP^-^ (*n*=3,154); ^b^HP^+^ (*n*=1,824), HP^-^ (*n*=3,153).
