# Supplementary material for: Anti-Helicobacter pylori antibody status is associated with cancer mortality: A longitudinal analysis from the Japanese DAIKO prospective cohort study
Source: PLOS Glob Public Health. 2023 Feb 8;3(2):e0001125. doi: 10.1371/journal.pgph.0001125 (PMC10022139; doi:10.1371/journal.pgph.0001125)
Supplement: S2 Table — (DOCX) [file pgph.0001125.s003.docx]

**S2 Table** **Continuous variables for participants after matching**

| Variable (unit) | HP^+^(*n*=1,688)  Median(1st-3rd quartile) | HP^-^(*n*=1,688)  Median(1st-3rd quartile) | *P* value |
| --- | --- | --- | --- |
| Age (yr) | 57.9 (48.6, 64.1) | 57.8 (48.8, 63.9) | 0.866 |
| Waist (cm) | 80.5 (74.6, 87) | 80 (73.9, 86) | 0.0592 |
| SBP (mmHg) | 117 (105.5, 132.5) | 116.5 (105.5, 131) | 0.664 |
| DBP (mmHg) | 71.5 (64, 81.5) | 72 (64.5, 81) | 0.862 |
| TC (mg/dL)^a^ | 210 (188, 235) | 211 (188, 235) | 0.983 |
| TG (mg/dL)^a^ | 81 (57, 117) | 79 (59, 114) | 0.907 |
| HDL (mg/dL)^a^ | 62 (52, 73) | 65 (54, 76) | 0.0000233 |
| AST (GOT) (IU/L)^a^ | 20 (17, 24) | 20 (17, 24) | 0.652 |
| ALT (GPT) (IU/L)^a^ | 16 (12, 21.5) | 16 (12, 21) | 0.883 |
| γ−GTP (IU/L)^a^ | 20 (14, 32) | 20 (14, 31) | 0.997 |
| Cr (mg/dL)^a^ | 0.6 (0.6, 0.7) | 0.6 (0.6, 0.7) | 0.836 |
| UA (mg/dL)^a^ | 4.7 (4, 5.6) | 4.6 (3.9, 5.6) | 0.144 |

HP, *Helicobacter* *pylori*; BMI, body mass index; SBP, systolic blood pressure; DBP, diastolic blood pressure; TC, total cholesterol; TG, triglycerides; HDL, high-density lipoprotein cholesterol; AST (GOT), aspartate aminotransferase (glutamic oxaloacetic transaminase); ALT (GPT), alanine aminotransferase (glutamic pyruvic transaminase). γ−GTP, gamma-glutamyl transpeptidase; Cr, creatinine; UA, uric acid. ^a^HP^+^ (*n*=1,687), HP^-^ (*n*=1,686).
