# Supplementary material for: Anti-Helicobacter pylori antibody status is associated with cancer mortality: A longitudinal analysis from the Japanese DAIKO prospective cohort study
Source: PLOS Glob Public Health. 2023 Feb 8;3(2):e0001125. doi: 10.1371/journal.pgph.0001125 (PMC10022139; doi:10.1371/journal.pgph.0001125)
Supplement: S3 Table — (DOCX) [file pgph.0001125.s004.docx]

**S3 Table** **Cancer incidence rate and all-cause deaths**

|  | Incidence per 1,000 person-year | |  |
| --- | --- | --- | --- |
|  | HP^+^ (n=1,688) | HP^-^ (n=1,688) | *P* value* |
| All cancer | 8.94 | 5.62 | 0.003 |
| Gastric | 1.66 | 0.41 | 0.003 |
| Non-Gastric** | 7.36 | 5.19 | 0.035 |
| Uterine (female only) | 1.05 | 0.57 | 0.275 |
| Lung | 0.50 | 0.66 | 0.597 |
| Prostate (male only) | 2.92 | 2.06 | 0.481 |
| Colon | 1.33 | 0.58 | 0.059 |
| Breast (female only) | 2.10 | 1.27 | 0.182 |
| Death | 2.23 | 2.22 | 0.990 |

*The p-value was calculated by the Wald test.

**Includes all patients who started with ICD-10 (except gastric).
