# Supplementary material for: Anti-Helicobacter pylori antibody status is associated with cancer mortality: A longitudinal analysis from the Japanese DAIKO prospective cohort study
Source: PLOS Glob Public Health. 2023 Feb 8;3(2):e0001125. doi: 10.1371/journal.pgph.0001125 (PMC10022139; doi:10.1371/journal.pgph.0001125)
Supplement: S4 Table — (DOCX) [file pgph.0001125.s005.docx]

**S4 Table** **Multivariate Cox regression models for all-cancer incidence (*n*=3,375)**

| Variable | HR | 95%CI Lower | 95%CI Upper | *P* value |
| --- | --- | --- | --- | --- |
| Age (yr) | 1.07 | 1.05 | 1.09 | 3.35 x 10^-11^ |
| Sex | 0.88 | 0.60 | 1.30 | 0.52 |
| Drinking | 1.24 | 0.88 | 1.74 | 0.216 |
| Smoking | 1.97 | 1.35 | 2.87 | 0.000422 |
| HP | 1.59 | 1.17 | 2.16 | 0.00297 |

HR, hazard ratio; HP, *Helicobacter pylori*.
