# Supplementary material for: Anti-Helicobacter pylori antibody status is associated with cancer mortality: A longitudinal analysis from the Japanese DAIKO prospective cohort study
Source: PLOS Glob Public Health. 2023 Feb 8;3(2):e0001125. doi: 10.1371/journal.pgph.0001125 (PMC10022139; doi:10.1371/journal.pgph.0001125)
Supplement: S5 Table — (DOCX) [file pgph.0001125.s006.docx]

**S5 Table** **Multivariate Cox regression models for gastric cancer incidence (*n*=3,375)**

| Variable | HR | 95%CI Lower | 95%CI Upper | *P* value |
| --- | --- | --- | --- | --- |
| Age | 1.11 | 1.04 | 1.19 | 0.000948 |
| Sex | 0.36 | 0.13 | 1.02 | 0.0542 |
| Drinking | 0.76 | 0.32 | 1.83 | 0.542 |
| Smoking | 1.40 | 0.50 | 3.90 | 0.519 |
| HP | 3.93 | 1.47 | 10.48 | 0.00631 |

HR, hazard ratio; HP, *Helicobacter pylori*.
