# Supplementary material for: Anti-Helicobacter pylori antibody status is associated with cancer mortality: A longitudinal analysis from the Japanese DAIKO prospective cohort study
Source: PLOS Glob Public Health. 2023 Feb 8;3(2):e0001125. doi: 10.1371/journal.pgph.0001125 (PMC10022139; doi:10.1371/journal.pgph.0001125)
Supplement: S6 Table — (DOCX) [file pgph.0001125.s007.docx]

**S6 Table** **Multivariate Cox regression models for non-gastric cancer incidence (*n*=3,375)**

| Variable | HR | *95%CI Lower* | *95%CI Upper* | *P* value |
| --- | --- | --- | --- | --- |
| Age | 1.06 | 1.04 | 1.09 | 5.02 x 10^-09^ |
| Sex | 0.99 | 0.66 | 1.50 | 0.975 |
| Drinking | 1.31 | 0.91 | 1.89 | 0.147 |
| Smoking | 2.09 | 1.40 | 3.13 | 0.0003 |
| HP | 1.42 | 1.02 | 1.97 | 0.0356 |

HR, hazard ratio; HP, *Helicobacter pylori*.
